# Zebrafish IL-4–like Cytokines and IL-10 Suppress Inflammation but Only IL-10 Is Essential for Gill Homeostasis

**DOI:** 10.4049/jimmunol.2000372

**Published:** 2020-08-07

**Authors:** Federica Bottiglione, Christopher T. Dee, Robert Lea, Leo A. H. Zeef, Andrew P. Badrock, Madina Wane, Laurence Bugeon, Margaret J. Dallman, Judith E. Allen, Adam F. L. Hurlstone

**Affiliations:** * Lydia Becker Institute of Immunology and Inflammation, School of Biological Sciences, Faculty of Biology, Medicine and Health, The University of Manchester, Manchester M13 9PT, United Kingdom; and; † Department of Life Sciences, Faculty of Natural Sciences, Imperial College London, London SW7 2AZ, United Kingdom

## Abstract

Mucosal surfaces such as fish gills interface between the organism and the external environment and as such are major sites of foreign Ag encounter. In the gills, the balance between inflammatory responses to waterborne pathogens and regulatory responses toward commensal microbes is critical for effective barrier function and overall fish health. In mammals, IL-4 and IL-13 in concert with IL-10 are essential for balancing immune responses to pathogens and suppressing inflammation. Although considerable progress has been made in the field of fish immunology in recent years, whether the fish counterparts of these key mammalian cytokines perform similar roles is still an open question. In this study, we have generated IL-4/13A and IL-4/13B mutant zebrafish (*Danio rerio*) and, together with an existing IL-10 mutant line, characterized the consequences of loss of function of these cytokines. We demonstrate that IL-4/13A and IL-4/13B are required for the maintenance of a Th2-like phenotype in the gills and the suppression of type 1 immune responses. As in mammals, IL-10 appears to have a more striking anti-inflammatory function than IL-4–like cytokines and is essential for gill homeostasis. Thus, both IL-4/13 and IL-10 paralogs in zebrafish exhibit aspects of conserved function with their mammalian counterparts.

## Key Points

- Zebrafish IL-4–like cytokines promote type 2 and suppress type 1 immunity.
- Zebrafish IL-10 is potently anti-inflammatory and essential for gill homeostasis.

## Introduction

In mammals, IL-4 and IL-13 are structurally and functionally related cytokines that participate in several physiological processes but are mainly known for stimulating type 2 immune responses (1), characterized by mucus and IgE production, eosinophilia, and differentiation of alternatively activated (M2) macrophages (2). Many aspects of type 2 immunity reinforce and protect barrier surfaces, including enhanced mucus production, rapid tissue repair, and IgE blockade of toxins (3–5). These responses confer protection against parasitic infection and some venoms but, when inappropriately activated, contribute to the development of asthma, allergic inflammation, and fibrosis (1, 6–8).

IL-4 or IL-13 or both are secreted by CD4^+^ Th2 cells, basophils, eosinophils, mast cells, and group 2 innate lymphoid cells (ILCs) but also by granulocytes associated with allergic responses, including basophils, eosinophils, and mast cells (9). IL-4 and IL-13 exert their functions by binding to two types of receptor complexes: a type I receptor, constituted by the IL-4Rα-chain and the IL-2R common γ-chain and a type II receptor, which comprises IL-4Rα-chain and IL-13Rα1 subunits. Type I receptor is mainly expressed in hematopoietic cells, particularly in lymphocytes, whereas the type II receptor is expressed on nonhematopoietic cells, such as epithelial cells. Interestingly, myeloid cells express both type I and type II receptors (1). Both type I and type II receptors signal through STAT6 transcription factor binding to promoter elements within IL-4/IL-13 responsive genes (10). IL-4 and IL-13 suppress inflammatory responses by antagonizing the production of TNF-α, IL-1β, and other proinflammatory mediators (11) and act in opposition to IFN-γ, the canonical Th1 (type 1) cytokine (12). Indeed, coordinately with inducing type 2 immune response, IL-4 and IL-13 suppress type 1 responses that are characterized by inflammation caused by immune cell–mediated destruction of cells infected with intracellular pathogens (13).

Although IL-4 and IL-13 can negatively regulate inflammatory responses, IL-10 has the more central anti-inflammatory role in mammals, potently suppressing IFN-γ responses. IL-10 can suppress a range of aberrant immune responses, including both type 1 and type 2 responses (14). IL-10 is produced by many cell types (15). It regulates CD4^+^ regulatory T cell differentiation and function, and it is important in maintaining homeostasis at mucosal surfaces (16). In the gastrointestinal tract, IL-10 regulates the immune response to the gut microbiota (17). A decrease of IL-10 production from intestinal epithelial cells leads to the development of gut inflammatory disorders (18). Moreover, lack of IL-10 induces the development of spontaneous inflammation at mucosal surfaces such as the lungs and intestine (19).

Evidence is emerging that fish immune responses can also be classified as type 1 or type 2 and that Th cells similar to mammalian counterparts likely do exist (20). Relevant cytokine receptors and downstream signaling pathway molecules are conserved between fish and mammals (21). However, there is still the need to understand how specific immune cells and cytokines function, in particular in mucosal tissues such as the gills. Fish gills represent an important mucosal surface that is constantly exposed to both waterborne pathogens and commensals and is able to mount immune responses (22, 23). Teleost gills are composed of four arches on each side of the head supported by cartilage or bone tissue. Each arch displays two files of filaments (primary lamellae). In turn, each filament is made of plate-like structures projecting on both sides (secondary lamellae) where gas exchange occurs (24). Lamellae are covered with a microridged respiratory epithelium, comprising pavement cells, mitochondria-rich chloride cells, and mucus-producing goblet cells (25). Several immune cells, including macrophages, neutrophils, and lymphocytes, are found dispersed within the lamellar epithelium, forming the so-called gill-associated lymphoid tissue (26). Moreover, an organized lymphoid area at the origin of the filaments, namely the interbranchial lymphoid tissue (ILT), has been described in salmonids (27, 28). ILT is rich in T cells that are embedded in a meshwork of epithelial cells (28), and it has been shown to play a role in maintaining immune tolerance and homeostasis in the gills (29), suggesting that the ILT represents a vital part of the gill-associated lymphoid tissue. A commensal microbial community has also been discovered in fish gills (30). As with all mucosal surfaces, the balance between inflammatory and regulatory responses is critical for the maintenance of tissue homeostasis and barrier integrity.

Two type 2 cytokines that are related to mammalian IL-4 and IL-13 have been described in teleost fish (31). It is likely that a single *il4/13* gene existed in ancestral jawed vertebrates, which has been duplicated in subsequent vertebrate lineages by whole genome duplication and/or tandem duplication events (32). Because of an additional round of whole genome duplication that occurred in fish, teleosts acquired two *il4/13* loci (ohnologues), namely *il4/13a* and *il4/13b* (33). Analysis of these loci shows that teleost *il4/13* genes share the following important characteristics with genes encoding tetrapod IL-4 and IL-13: their positioning relative to *rad50* and *kif3a* gene neighbors (34), the typical short-chain type 1 cytokine organization, and conserved structural motifs (33). The identification of putative binding motifs for GATA3, a transcription factor that directs mammalian Th2 and ILC2 development (35), in the promoter regions of teleost *il4/13* genes further supports that these genes encode Th2 cytokines (33).

Enriched levels of mRNA encoding IL-4/13A together with the transcription factor GATA3 have been detected in salmonid fish mucosal tissues, such as the gills and the skin, indicating these to represent Th2-skewed environments that protect fish from parasites and inflammatory responses (36). We have also identified a population of CD4^+^ Th2-like lymphocytes and their signature cytokines in zebrafish gills (37). To date, a comprehensive characterization of fish IL-4/13 paralogs is lacking, and whether *il4/13a* and *il4/13b* represent authentic *IL-4* and *IL-13* orthologs is still a matter of debate. IL-10 has been described in several fish species (38–41), and its constitutive expression was reported in zebrafish kidney, gut, and gills (41), suggesting a role in maintaining homeostasis in these tissues. Exploring the conservation and divergence of IL-4/13 and IL-10 cytokines would further our understanding of fish immune responses and may also provide an alternative model for dissecting aspects of mammalian immunity.

In this study, we used zebrafish to study the functions of fish IL-4/13A, IL-4/13B, and IL-10 cytokines. We generated zebrafish knockouts for *il4/13a* and *il4/13b* genes and addressed the effects of their downregulation in both larvae and adult fish immunity. We showed the importance of IL-4/13A and IL-4/13B in suppressing inflammation as well as maintaining a Th2 phenotype in the gills. To gain further insight into the regulation of inflammation, the gills of *il10*-deficient zebrafish were examined, revealing the requirement for IL-10 in maintaining homeostasis in this mucosal tissue.

## Materials and Methods

### Zebrafish care

Zebrafish (*Danio rerio*) were maintained under standard conditions (∼28°C under a 14-h light/10-h dark cycle) within the Biological Services Unit (The University of Manchester) and the Central Biomedical Services (Imperial College London). Embryos were collected and raised in egg water (Instant Ocean Salt 60 μg/ml) up to 5 d postfertilization (dpf) and then transferred to the main aquarium system. Younger fry were fed powder food and rotifers, whereas older fry and adults were fed powder food and brine shrimp. All food was supplied by Zebrafish Management fish food. *il10^e46/e46^* mutant zebrafish were a kind gift from S. Johnston (University of Sheffield) and were generated by the Sanger Institute through the Zebrafish Mutation Project (42). All regulated procedures received ethical approval from the institutions’ ethical review boards and were performed under Home Office License (project licenses PF74F0848 and P5D71E9B0), according to the United Kingdom’s Animal Act.

### CRISPR/Cas9-mediated generation of mutant lines

#### Creation of single chimeric guide RNA targeting il4/13a and il4/13b.

Guide RNAs (gRNAs) were designed to target the first exon of either *il4/13a* or *il4/13b* zebrafish genes using the Harvard chopchop program (https://chopchop.rc.fas.harvard.edu). The *il4/13a* target site was 5′-GGGTTTTACGTTGAAAGGCA-3′, and the *il4/13b* target site was 5′-GAAATCATCCAGAGTGTGAA-3′ and were incorporated into a gRNA template for transcription using PCR. The forward primer contained the target gene specific sequence followed by a constant sequence that overlaps with the remaining sequence of *Streptococcus pyogenes* chimeric gRNA DNA template (plasmid no. 51132; Addgene), whereas the reverse primer was complementary to the gRNA DNA template (see

**Table I.** List of primers used to generate the single gRNA templates

Name	Forward	Reverse
*il4/13a*	5′-TAATACGACTCACTATAGGGTTTTACGTTGAAAGGCAGTTTTAGAGC-3′	5′-AAAAGCACCGACTCGGTGCCACTTTTTCAAG-3′
*il4/13b*	5′-TAATACGACTCACTATAGAAATCATCCAGAGTGTGAAGTTTTAGAGC-3′	5′-AAAAGCACCGACTCGGTGCCACTTTTTCAAG-3′

Table I for primer sequences). PCR amplification of the gRNA DNA template using a high fidelity Phusion Taq polymerase was performed, and PCR products were gel purified and used for the synthesis of gRNAs using an Ambion MEGAshortscript T7 kit, according to the manufacturer’s instructions.

#### Injecting CRISPR reagents.

Injection solutions included 30 pg/nl of either gRNA, 250 pg/nl of codon-optimized *S. pyogenes* nls-zCas9-nls mRNA synthesized using a mMESSAGE mMACHINE SP6 Kit (Life Technologies) from a pCS2 construct (plasmid no. 47929; Addgene), 100 pg/nl H2B-mCerulean3 tracer mRNA similarly generated from a pCS2 construct, and 0.05% (w/v) phenol red to allow visualization of injections. Embryos were injected at the one-cell stage and screened for fluorescence at 24 h postfertilization to identify positively injected fish, which were then raised to adulthood.

#### Identifying and raising mutant lines.

To identify modified alleles in the F1 progeny for the establishment of *il4/13a* and *il4/13b* zebrafish mutant lines, genomic DNA was amplified by PCR using gene-specific primers flanking the targeted site (see

**Table II.** List of primers used for genotyping PCR

Name	Forward	Reverse
*il4/13a*	5′-GCACTGTATTCGTCTCGGGTTTTA-3′	5′-TTTTCCCCAGATCTACAAGGAAGA-3′
*il4/13b*	5′-CTGTTGGTACTTACATTGGTCCCC-3′	5′-AGTGTCCTGTCTCATATATGTCAGGT-3′

Table II for primer sequences), and abnormal sized products were gel purified. One microliter of sample was cloned into a TOPO cloning vector (Invitrogen). Cloning reactions were performed according to the manufacturer’s instructions. Inserts were sequenced by Sanger sequencing using Eurofins Genomics services. Fish carrying selected mutations were out crossed and their F2 progeny genotyped for the presence of mutations (see

**Table III.** List of primers used for qPCR

Name	Forward	Reverse
*β actin*	5′-CGAGCTGTCTTCCCATCCA-3′	5′-TCACCAACGTAGCTGTCTTTCTG-3′
*tnfa*	5′-ACAAGATGGAAGTGTGCTGAGA-3′	5′-ATTTCAAGCCACCTGAAGAAAA-3′
*il6*	5′-CCTCTCCTCAAACCTTCAGACC-3′	5′-TGCTGTGTTTGATGTCGTTCAC-3′
*il1b*	5′-GGACTTCGCAGCACAAAATGAA-3′	5′-TTCACTTCACGCTCTTGGATGA-3′
*il4/13b*	5′-GCAGGAATGGCTTTGAAGGGTAAA-3′	5′-AAACTCCTTCATTGTGCATTCCCC-3′
*il4/13a*	5′-GCACTGTATTCGTCTCGGGTTTTA-3′	5′-TTTTCCCCAGATCTACAAGGAAGA-3′
*il10*	5′-CTTTAAAGCACTCCACAACCCCAA-3′	5′-CTTGCATTTCACCATATCCCGCTT-3′
*mpx*	5′-GAGCTGCAGACTACATGGCACA-3′	5′-CTAGCCACTGCAGATGCTGACATAG-3′
*ifng1.2*	5′-CCTGGGGAGTATGTTTGCTGTTTT-3′	5′-GGGTGTGCATTATGTAGCTGAGAA-3′

Table II for primer sequences). *Il4/13a*^+/−^ and *il4/13b*^+/−^ heterozygous fish were in crossed to generate homozygous mutants for both *il4/13a* (*il4/13a*^−/−^) and *il4/13b* (*il4/13b*^−/−^) in the F3 generation. To generate *il4/13a;b*^−/−^ double mutants, *il4/13a*^−/−^ and *il4/13b*^−/−^ homozygous fish were intercrossed and their progeny in crossed. *il4/13a;b*^−/−^ double mutants were in crossed to maintain the line.

### Whole mount in situ hybridization

Three-day-old embryos (*n* = 9–10) were fixed in 4% (w/v) paraformaldehyde in PBS at 4°C overnight. Fish were then washed in 0.1% (v/v) Tween 20 PBS solution and dehydrated through an ascending series of methanol. Digoxigenin-labeled RNA antisense probe for zebrafish *mpx* was kindly supplied by S. Renshaw (University of Sheffield), and whole mount in situ hybridization was performed as described in (43). Embryos fixed in glycerol were mounted on a glass slide, and images were collected on a Leica MZ16FA stereomicroscope equipped with a DC490 camera. Quantification of *mpx*^+^ cells was performed using ImageJ software.

### Real-time quantitative PCR

RNA was isolated from homogenized larvae and/or adult gills (*n* > 3) using the RNeasy Mini Kit (QIAGEN) and reverse transcribed using the ProtoScript II First Strand cDNA Synthesis Kit (New England BioLabs) with Oligo (dT) primers, according to the manufacturer’s instructions. Quantitative PCR (qPCR) was performed using SYBR Green JumpStart Taq ReadyMix (Sigma), ROX Reference Dye, and cDNA (diluted 1:2). All samples were run in triplicate on 96-well PCR plates (BioLabs). Negative controls were run without cDNA. Data were analyzed by the ΔCt method using *bactin* for normalization. The primers used for qPCR are listed in Table III.

### Histology

Whole heads were collected from wildtype (*n* = 3), *il4/13a^−/−^* (*n* = 4), *il4/13b^−/−^* (*n* = 4), *il4/13a;b^−/−^* (*n* = 4), and *il10^e46/e46^* (*n* = 6) as well as wildtype and *il10^e46/e46^* animals following 8-h resiquimod (R848) challenge (*n* = 3) and fixed in 4% (w/v) paraformaldehyde overnight at 4°C. The next day, the samples were washed in PBS and decalcified in 0.25 M EDTA solution for 3 d at room temperature. After decalcification, heads were washed with tap water and placed in 70% (v/v) EtOH. All specimens were dehydrated, and paraffin wax embedded. Sections (5 μm) were cut on a Leica rotary microtome RM2235 and mounted on slides (SuperFrost Plus, Menzel-Glaser; Thermo Fisher Scientific). Sections (*n* = 3) were deparaffinized, rehydrated, and stained with H&E. Staining and coverslipping was performed on a Leica workstation operated by the Histology Facility at The University of Manchester. Slides were scanned on a slide scanner (Pannoramic P250 Flash III), and images were processed using 3DHISTECH software. Gill tissue damage was quantified using a score adapted from Mitchell et al. (44) ranging from 0 to 3, where 0 indicates the absence of lamellar fusion, epithelial hyperplasia, or altered tissue architecture, 1 indicates <10% of gill tissue affected, 2 indicates 10–50% of gill tissue affected, and 3 indicates >50% of gill tissue affected.

### RNA sequencing

Gills were dissected from 6-mo-old fish; RNA was isolated and subjected to sequencing analysis. Prior to performing RNA sequencing (RNA-seq), the integrity of RNA samples was assessed using a 2200 TapeStation (Agilent Technologies), according to the manufacturer’s instructions. Total RNA was submitted to the Genomic Technologies Core Facility of the University of Manchester, and libraries were generated using the TruSeq Stranded mRNA assay (Illumina), according to the manufacturer’s protocol. Adapter indices were used to multiplex libraries, which were pooled prior to cluster generation using a cBot instrument. The loaded flow cell was then paired-end sequenced (76 + 76 cycles, plus indices) on an Illumina HiSeq 4000 instrument. Finally, the output data were demultiplexed (allowing one mismatch) and BCL-to-Fastq conversion performed using Illumina’s bcl2fastq software (version 2.17.1.14). Unmapped paired-end sequences were tested by FastQC (http://www.bioinformatics.babraham.ac.uk/projects/fastqc/). Sequence adapters were removed, and reads were quality trimmed using Trimmomatic_0.36 (45). The reads were mapped against the reference zebrafish genome (danRer10), and counts per gene were calculated using the annotation from Ensembl (http://ftp.ensembl.org/pub/current_gtf/danio_rerio/Danio_rerio.GRCz10.84.gtf.gz) using STAR_2.5.3 (46). The RNA-seq datasets reported in this paper have been deposited in the ArrayExpress database (https://www.ebi.ac.uk/arrayexpress/; accession number E-MTAB-8958). Normalization, principal component analysis (PCA), and differential expression were identified using DESeq2_1.16.1 (47) as genes having a corrected *p* value <0.05 and log_2_ fold change >|1|. Venn diagrams were generated using Venny 2.1.0 (48) to identify the presence of common upregulated (log_2_ fold change > 1) and downregulated (log_2_ fold change < −1) genes in *il4/13a^−/−^*, *il4/13b^−/−^*, and *il4/13a;b^−/−^* gills.

### Functional analysis of differentially expressed genes

Cluster analysis was performed using k-means clustering (Manhattan distance) using maxdView software. Each cluster was then hierarchically clustered. Clustering was performed on the means of each sample group (log 2) that had been *z*-transformed (for each gene, the mean set to zero, SD to one). Gene ontology analysis and pathway analysis were performed with Enrich (49) using human orthologs of zebrafish genes obtained from Ensembl genome browser version 99. Gene set enrichment analysis (GSEA) was performed using GSEA software (50). Several gene sets consisting of different immune cell markers, spanning innate and adaptive immune cell types, were generated using the human immunology panel from NanoString Technologies. Normalized enrichment scores (NES) were used to generate heatmaps with Morpheus software (https://software.broadinstitute.org/morpheus). xCell analysis was used to perform cell type enrichment analysis from gene expression data for 64 immune cell types (https://xcell.ucsf.edu).

### R848 gill challenge

R848 gill challenge was performed as previously described (51) using 5 μl of R848 (0.5 mg/ml; InvivoGen) applied to the gills for 5 min. Following completion of the procedure, fish were returned to fresh system water and monitored for their recovery. Fish were then sacrificed with an overdose of MS222 (800 mg/l) at the indicated timepoint to harvest the gills for RNA extraction.

### Statistical analysis

Statistical analysis was performed using GraphPad Prism version 8 (GraphPad Software). The *p* values <0.05 (where necessary, corrected for multiple testing) were considered statistically significant. Throughout, error bars represent SEM.

## Results

### Generation of il4/13a and il4/13b zebrafish mutants

**FIGURE 1.**
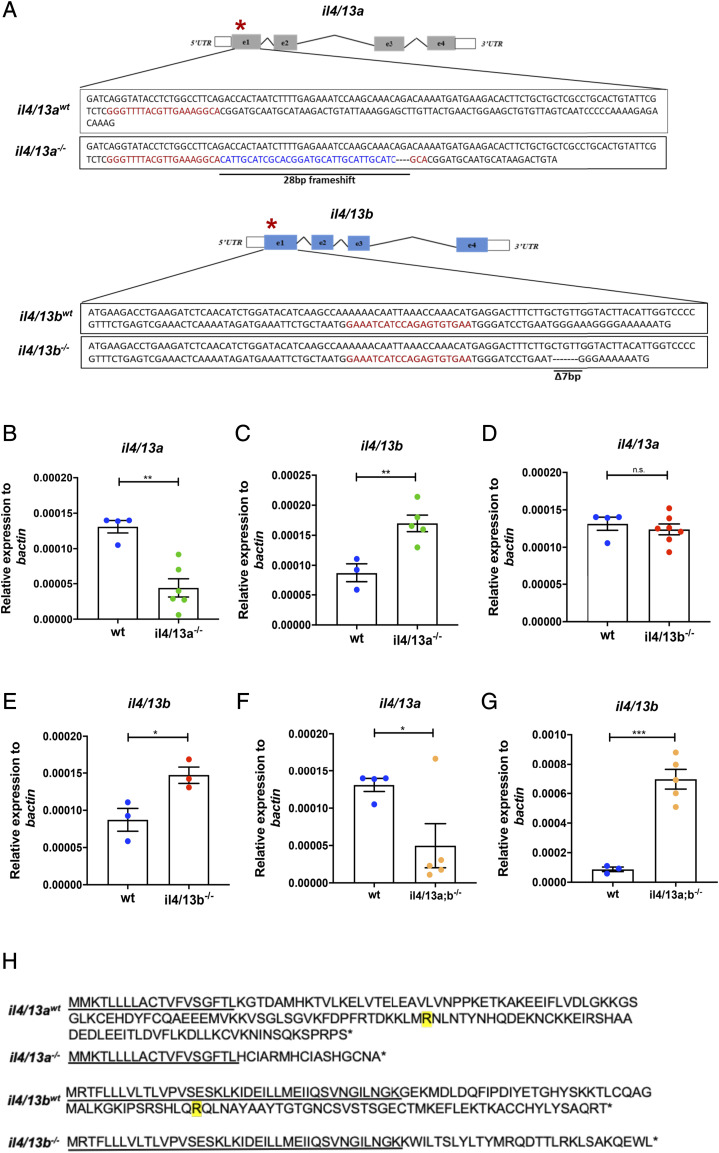
Generation of *il4/13a^−/−^* and *il4/13b^−/−^* zebrafish mutants by CRISPR/Cas9. (**A**) Zebrafish *il4/13a* and *il4/13b* genes. (**B**–**G**) qPCR analysis showing the levels of *il4/13a* and *il4/13b* transcripts in wildtype, *il4/13a^−/−^*, *il4/13b^−/−^*, and *il4/13a;b^-^*^/-^ 5 dpf larvae. Gene expression was normalized to the expression of β−actin. Error bars represent SEM; *n* > 3, *p < 0.05, **p < 0.01, ***p < 0.001. (**H**) Sequence of wildtype and mutated IL-4/13A and IL-4/13B proteins. Underlined is the unchanged portion of the protein sequence in mutant animals. Yellow-shaded “R” indicates the conserved arginine residue required in the human homologs for receptor binding. The asterisks indicate a stop codon.

In zebrafish, two similar *il4*-like genes, namely *il4/13a* and *il4/13b*, have been described (33). To generate insight into the function of these two genes, we used CRISPR technology to create mutant lines lacking functional alleles. An *il4/13a* allele carrying a 28-bp frameshift mutation (32-bp insertion and 4-bp deletion) and an *il4/13b* allele carrying a 7-bp deletion were isolated (Fig. 1A). Homozygous mutants for both *il4/13a* (*il4/13a*^−/−^) and *il4/13b* (*il4/13b*^−/−^) were found to be viable, and they were intercrossed to generate *il4/13a;b*^−/−^ double mutants. These were also found to be viable and fertile.

To evaluate whether the mutations affected cognate mRNA levels, qPCR analysis was performed. A significant reduction of *il4/13a* mRNA in *il4/13a^−/−^* embryos was observed (Fig. 1B), suggesting mRNA degradation by the nonsense-mediated decay pathway (52). Perhaps as a consequence of nonsense-mediated decay, an increase of *il4/13b* mRNA (1.6-fold) was detected in *il4/13a*^−/−^ embryos (Fig. 1C), suggesting a potential mechanism of genetic compensation, as recently described (53). No changes in the levels of *il4/13a* mRNA were observed in *il4/13b^−/−^* larvae (Fig. 1D). The expression of *il4/13b* mRNA was found increased in *il4/13b^−/−^* larvae (Fig. 1E), suggesting that the effects of the mutation might only be evident at the translational level and that increased mRNA might be an attempt to compensate for loss of function of the IL-4/13B protein. qPCR analysis also revealed that the expression of *il4/13a* was significantly reduced in *il4/13a;b*^−/−^ embryos (Fig. 1F), whereas the expression of *il4/13b* was significantly increased (Fig. 1G), in agreement with what was observed in the single mutants.

The selected mutations were predicted to introduce a premature termination codon in both *il4/13a* and *il4/13b* genes, leading to the formation of severely truncated proteins. Indeed, only 16 and 28 aa remained in the truncated IL-4/13A and IL-4/13B proteins, respectively (Fig. 1H). Previous analysis of zebrafish protein sequences revealed conservation of an arginine in the αC helix (54), which is critical for the binding of human IL-4 to the IL-4Rα receptor. The mutations identified in IL-4/13A and IL-4/13B proteins abolished this residue, implying that the proteins should not be able to bind to their receptors and thus lack any functional activity.

### Disruption of both il4/13a and il4/13b genes leads to a proinflammatory phenotype in larvae

**FIGURE 2.**
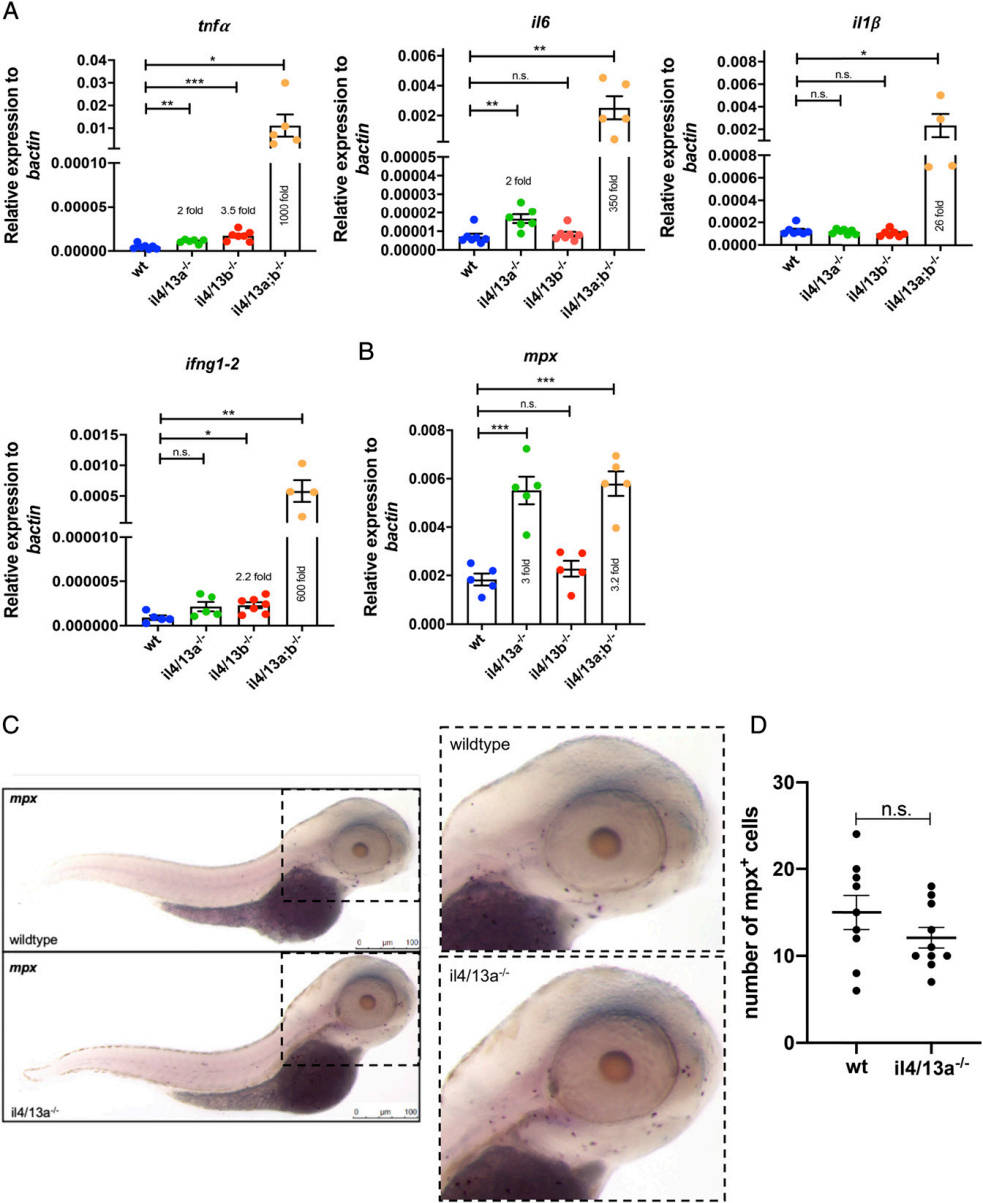
Increased levels of proinflammatory cytokines in *il4/13a;b^−/−^* larvae. (**A** and **B**) qPCR analysis showing levels of *il1β*, *il6*, *tnfα*, *ifng1–2*, and *mpx* mRNA in wildtype, *il4/13a^−/−^*, *il4/13b^−/−^*, and *il4/13a;b^−/−^* 5 dpf larvae. Dots indicate individual fish. Gene expression was normalized to the expression of β−actin. Error bars represent SEM; *n* > 3, *p < 0.05, **p < 0.01, ***p < 0.001. (**C**) Representative image of *mpx* in situ hybridization in wildtype and *il4/13a^−/−^* 3 dpf larvae (*n* = 9–10). Purple dots indicate *mpx^+^* cells. Scale bar, 100 μm. (**D**) Quantification of *mpx^+^* cells in wildtype and *il4/13a^−/−^* larvae head region. Dots indicate individual fish.

The functions of *il4/13a* and *il4/13b* genes were first investigated in larvae, which possess an innate immune system only (55). Whereas a modest increase of *tnfα*, *il6*, and *ifng1–2* mRNA encoding proinflammatory cytokines was observed in *il4/13a*^−/−^ and *il4/13b*^−/−^ single mutants, more pronounced upregulation was observed in *il4/13a;b*^−/−^ double mutants (Fig. 2A), indicating that *il4/13a* and *il4/13b* genes have a fundamental albeit redundant role in suppressing inflammation.

Next, we investigated whether the inflammatory phenotype was accompanied by an increase of neutrophils by measuring the levels of myeloperoxidase *mpx* transcript in larvae. A significant upregulation of *mpx* was detected in *il4/13a;b*^−/−^ double mutants (3.2-fold), supporting the inflammatory phenotype. A similar increase of *mpx* was observed in *il4/13a*^−/−^ larvae (3-fold) but not in *il4/13b*^−/−^ larvae (Fig. 2B), suggesting the increase in *mpx* transcript might be due to loss of functional IL-4/13A. Thus, in situ hybridization for *mpx* mRNA was performed on wildtype and *il4/13a*^−/−^ larvae to further investigate changes in neutrophils. However, no difference in neutrophil numbers or distribution were detected in *il4/13a*^−/−^ embryos (Fig. 2C, 2D).

### Transcriptome analysis of the gills reveals a shift toward type 1 immunity in il4/13a;b^−/−^ mutants

Our previous work demonstrated the expression of *il4/13a* in the gill tissue of adult zebrafish as well as *il4/13b* expression in gill-resident CD4^+^ T cells, suggesting the presence of a Th2-skewed environment in the gills (37). To investigate the gene expression profile of gills from *il4/13a^−/−^*, *il4/13b^−/−^*, and *il4/13a;b^−/−^* mutant animals, RNA-seq was performed on RNA isolated from the gills of adult fish. An overview of the RNA-seq analysis pipeline is shown in Supplemental Fig. 1A. PCA revealed clear segregation and clustering of mRNA profiles according to genotype (Supplemental Fig. 1B). Significantly differentially expressed genes were identified in the gills of mutant animals compared with wildtype (Supplemental Table I). Venn diagram analysis revealed numerous genes uniquely affected by IL-4/13a or IL-4/13b loss, fewer common targets affected by loss of either, and a significant number of redundant targets only affected upon loss of both cytokines (Supplemental Fig. 1C). To identify coordinated changes in gene expression within our samples, hierarchical clustering was performed. Differentially expressed genes were grouped in 10 clusters that were subjected to gene ontology analysis using Enrichr. Enriched gene ontologies were mainly associated with immune-related biological processes (Supplemental Fig. 2).

**FIGURE 3.**
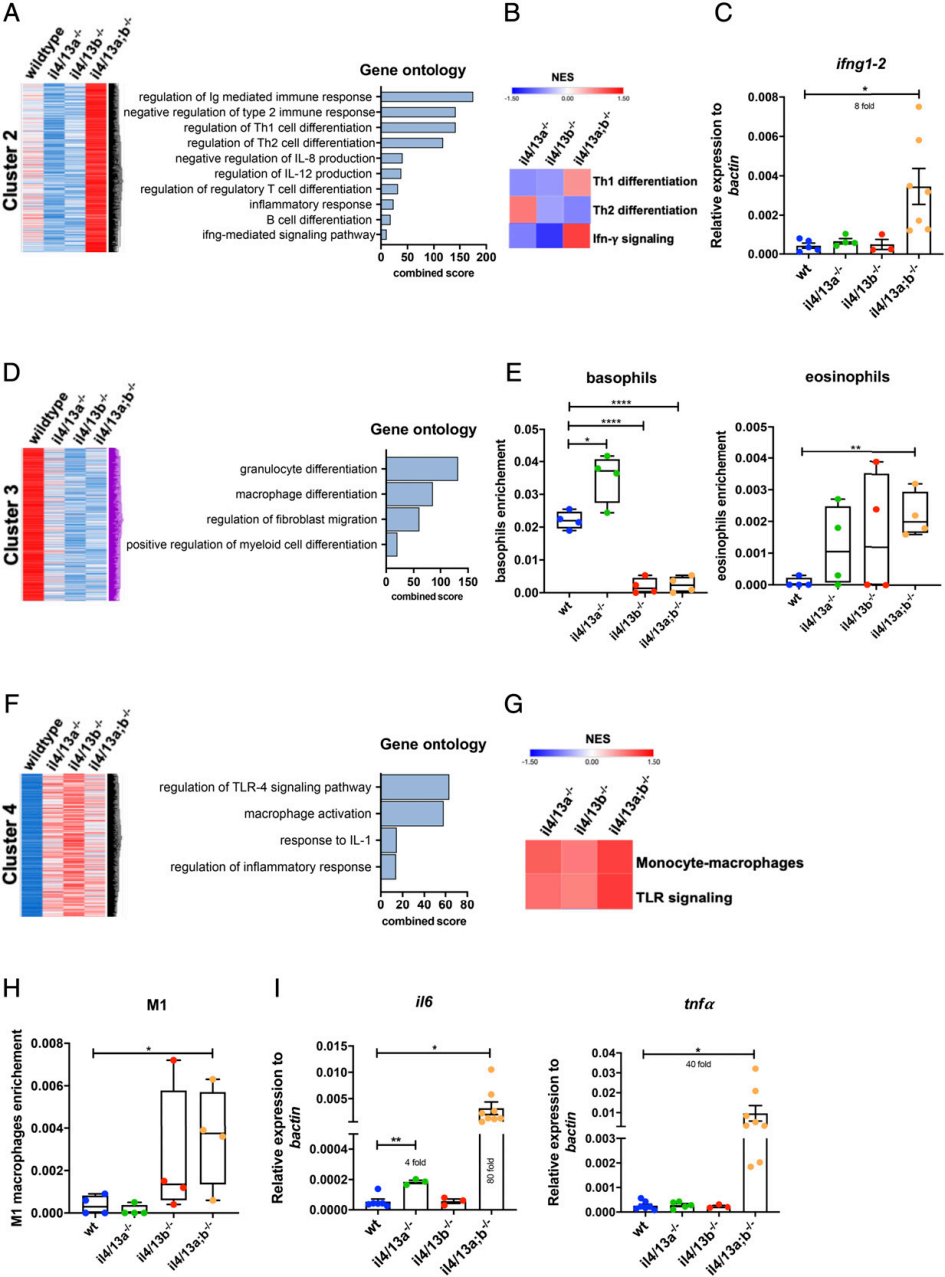
Transcriptome analysis of *il4/13a^−/−^*, *il4/13b^−/−^*, and *il4/13a;b^−/−^* gills. (**A**, **D**, and **F**) Clustering of differentially expressed genes identified in gills harvested from 6-mo-old fish. Blue indicates low expression, red indicates high expression, and white indicates unchanged expression. Bar graphs show significantly enriched ontologies for each cluster of genes. (**B** and **G**) Heatmap of NES for the indicated gene sets generated by GSEA in mutant gills compared with wildtype gills. (**C** and **I**) qPCR analysis showing transcript levels of *ifng1–2*, *il6*, and *tnfa* in wildtype and mutant gills. Gene expression was normalized to the expression of β−actin. Error bars represent SEM; *n* > 3, *p < 0.05, **p < 0.01, ****p < 0.0001. (**E** and **H**) Enrichment score of basophils, eosinophils, and M1 macrophages obtained by xCell analysis.

To characterize the transcriptional profile of *il4/13a;b*^−/−^ double mutants, we focused on the genes belonging to cluster 2 that were more highly expressed in *il4/13a;b*^−/−^ double mutants than any other genotype. Gene ontology analysis revealed that these genes were mainly associated with the negative regulation of type 2 immune responses, Th1 differentiation, and IFN-γ signaling (Fig. 3A). GSEA corroborated the inference that enhanced Th1 differentiation, diminished Th2 differentiation, and increased IFN-γ signaling was occurring in the gills of *il4/13a;b*^−/−^ double mutants (Fig. 3B). Moreover, qPCR confirmed increased levels of *ifng1–2* mRNA in the gills of *il4/13a;b*^−/−^ double mutants (Fig. 3C). All together, these data suggest that in the absence of both *il4/13a* and *il4/13b*, there might be a shift toward type 1 immunity, comparable to the role of IL-4 in mammals.

Next, we sought to investigate the gene signature of cluster 3 as these genes were downregulated in gills from *il4/13a^−/−^*, *il4/13b^−/−^*, and *il4/13a;b^−/−^* animals. Gene ontology analysis revealed that genes from cluster 3 were mainly associated with granulocyte differentiation, differentiation of myeloid cells, and regulation of fibroblast migration (Fig. 3D). To characterize potential enrichment of immune cell populations, xCell analysis was performed. Among granulocytes, basophils were predicted to be significantly reduced in gills from *il4/13b*^−/−^ single mutant and *il4/13a;b*^−/−^ double mutants, whereas an increase of eosinophils was predicted for *il4/13a;b*^−/−^ double mutants at least (Fig. 3E).

Genes belonging to cluster 4 were found highly upregulated in gills from *il4/13a*^−/−^, *il4/13b*^−/−^, and *il4/13a;b*^−/−^ animals, and they were found associated with processes such as TLR4 signaling, macrophage activation, response to IL-1, and regulation of the inflammatory response (Fig. 3F). Accordingly, an enrichment of monocytes–macrophage expression profile as well as TLR signaling signature was detected by GSEA (Fig. 3G). Moreover, xCell analysis predicted an increase of M1 macrophages in the gills of *il4/13a;b*^−/−^ double mutants (Fig. 3H). To further validate these data, we measured the expression of proinflammatory cytokines in the gills by qPCR and found an increased expression of *il6* (80-fold) and *tnfα* (40-fold) mRNA in the gills of *il4/13a;b*^−/−^ double mutants (Fig. 3I), suggesting overall heightened inflammatory activity.

### Loss of il4/13a and il4/13b does not affect gill morphology, whereas lack of functional il10 does

**FIGURE 4.**
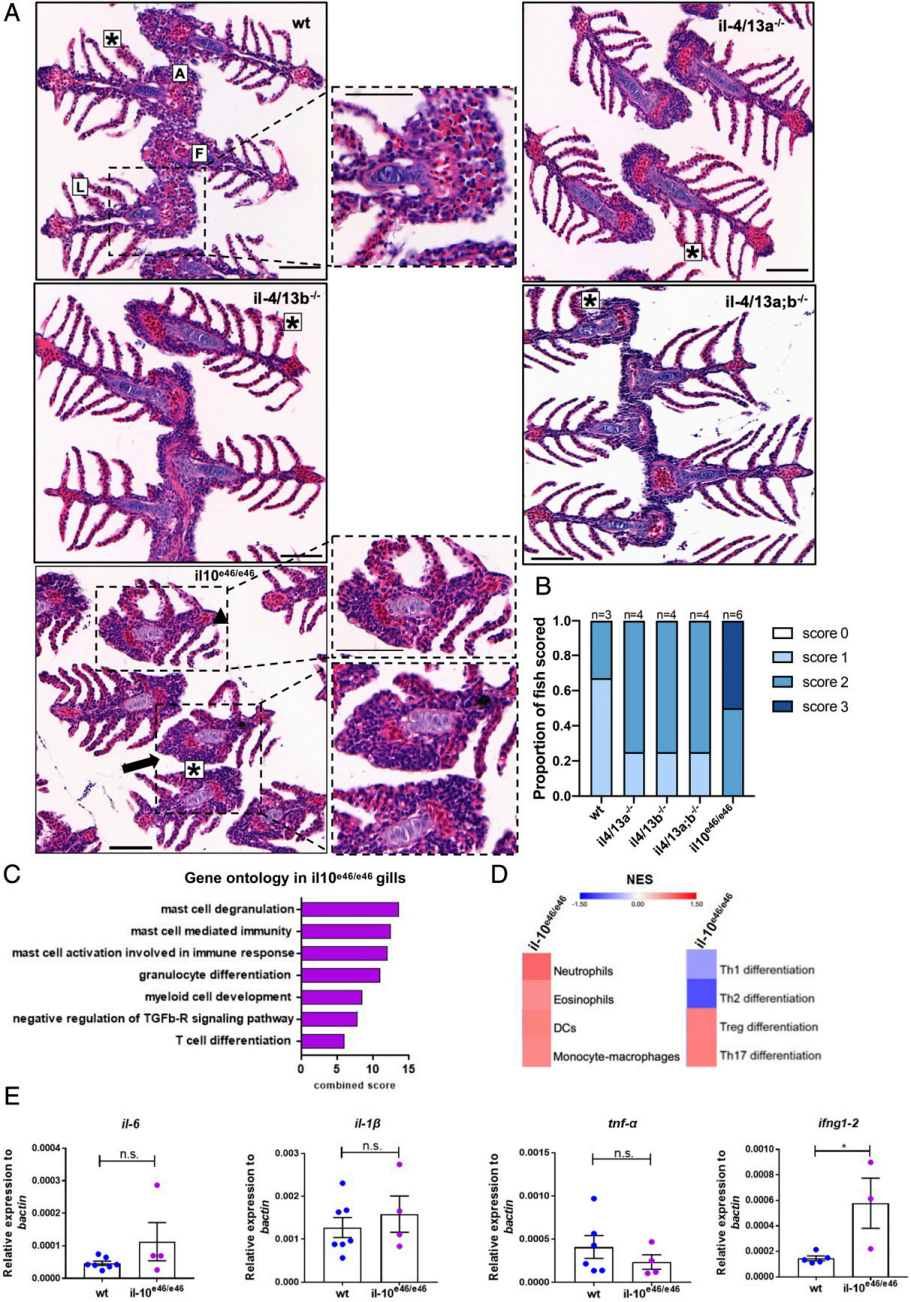
Mutation of *il10* induces morphological changes in the gills of adult zebrafish. (**A**) Representative images of sections (*n* = 3) of gills from wildtype, *il4/13a*^−/−^, *il4/13b*^−/−^, *il4/13a;b*^−/−^, and *il10^e46/e46^* animals stained with H&E. Gill arch (A), filaments (F), and lamellae (L) are indicated. Lamellar epithelial cell alteration (asterisks), filament thickening (triangle), and arch thickening (arrow) are indicated. (**B**) Quantification of gill tissue damage in wildtype, *il4/13a*^−/−^, *il4/13b*^−/−^, *il4/13a;b*^−/−^, and *il10^e46/e46^*. Widespread alteration is observed in the gills of *il10^e46/e46^* animals. (**C**) Bar graph showing significantly enriched ontologies in gills of *il10^e46/e46^* animals. (**D**) Heatmap of NES for indicated gene sets generated by GSEA in gills of *il10^e46/e46^* compared with wildtype animals. (**E**) qPCR analysis showing transcript levels of *il1β*, *il6*, *tnfα*, and *ifng1–2* in gills of *il10^e46/e46^* compared with wildtype animals. Gene expression was normalized to the expression of β−actin. Error bars represent SEM; *n* > 3, *p < 0.05.

To further evaluate the effects of *il4/13a* and *il4/13b* on zebrafish gill homeostasis, histology was performed. No significant morphological changes were observed in the gills of *il4/13a^−/−^*, *il4/13b^−/−^*, or *il4/13a;b^−/−^* fish compared with wildtype (Fig. 4A), suggesting that loss of functional IL-4/13A and IL-4/13B does not affect tissue integrity despite a shift toward type 1 immunity. For comparison, we examined the gills of *il10^e46/e46^* mutants lacking functional IL-10, which have also been reported to shift toward type 1 immune responses (56). A thickening of the gill arch and filaments, as well as lamellar epithelial changes, were detected in the absence of functional IL-10 (Fig. 4A, 4B), indicating a role for this cytokine in maintaining gill homeostasis in adult zebrafish.

To further investigate the role of *il10* in gill homeostasis, RNA-seq was performed on RNA extracted from the gills of *il10^e46/e46^* mutant animals. Again, PCA confirmed a distinct expression profile for *il10^e46/e46^* mutant animals (Supplemental Fig. 1B). Gene ontology analysis was performed using Enrichr, and an enrichment of immune-related ontologies, particularly related to mast cell and granulocyte differentiation, was observed (Fig. 4C). We also performed pathway analysis using Enrichr and found further evidence for an ongoing inflammatory response, highlighting the action of the complement system, chemokine and cytokine signaling, and other mediators of inflammation (Table IV). To investigate potential enrichment by immune cell populations, GSEA was performed. Enrichment of neutrophils, eosinophils, dendritic cells, and monocyte–macrophages was predicted in gills from *il10^e46/e46^* mutant animals. Moreover, a depletion of Th1 and Th2 cells but an enrichment of regulatory T cells and Th17 was also predicted (Fig. 4D). To validate these data, we measured gene expression changes by qPCR analysis. No significant changes were observed in the levels of *il1β*, *tnfα*, and *il6* mRNA encoding inflammatory cytokines but an increase of *ifng1–2* mRNA was detected in *il10^e46/e46^* gills compared with wildtype (Fig. 4E).

**Table IV.** Significantly enriched pathways in the gills of *il10*^e46/e46^ animals

*il-10*^e46/e46^ Gills	*p* Value	Combined Score
Hematopoietic cell lineage (Kyoto Encyclopedia of Genes and Genomes)	0.009404725	21.44861305
Cells and molecules involved in local acute inflammatory response (WP4493)	0.038843362	11.08262125
Human complement system (WP2806)	0.002197324	5.694253785
Inflammation mediated by chemokine and cytokine signaling pathway (Panther)	0.024404585	5.291463582
IL-23–mediated signaling events (National Cancer Institute)	0.030898252	4.721254283
Cell to cell adhesion signaling (BioCarta)	0.020030828	3.088771356

Combined scores and *p* values are indicated.

### Loss of IL-4/13a and IL-4/13b leads to an enhanced type 1 response to R848 in the gills of adult zebrafish

**FIGURE 5.**
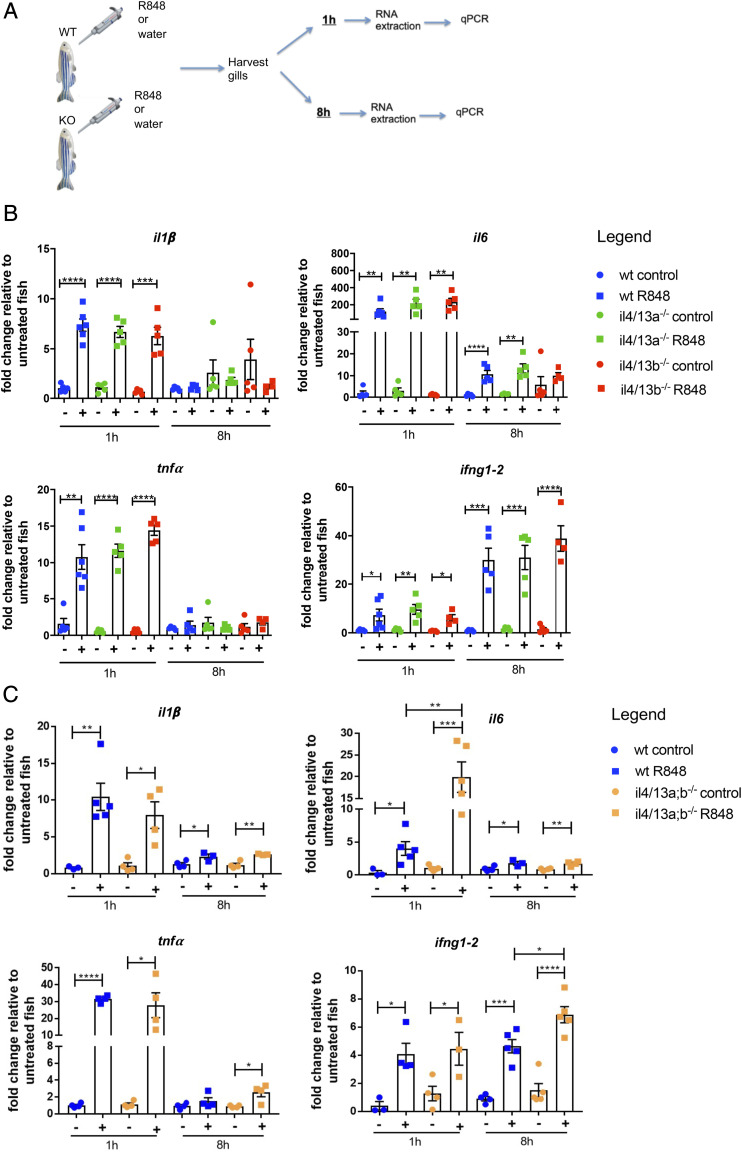
R848 induced an enhanced type 1 response in the gills of *il4/13a;b^−/−^* double mutants. (**A**) Schematic overview of R848 gill challenge. (**B** and **C**) qPCR analysis showing transcript levels of *il1β*, *il6*, *tnfα*, and *ifng1–2* in gills from wildtype, *il4/13a^−/−^*, *il4/13b^−/−^*, and *il4/13a;b^−/−^* animals following R848 challenge for 1 and 8 h. Gene expression was normalized to the expression of β−actin. Fold change is shown. Error bars represent SEM; *n* > 3, *p < 0.05, **p < 0.01, ***p < 0.001, ****p < 0.0001.

We next sought to investigate the importance of IL-4/13a and IL-4/13b in modulating an inflammatory response in the gills to an exogenous source of irritant. To induce gill inflammation, we used an intervention we have recently established, whereby a solution of R848, a synthetic compound that mimics viral ssRNA and interacts with TLR7/8 in mammals (57), is applied directly to the gills (51). Gene expression was analyzed by qPCR at 1 and 8 h to evaluate the kinetics of the response (Fig. 5A). An inflammatory response was observed in the gills of wildtype, *il4/13a^−/−^*, and *il4/13b^−/−^* animals after 1 h as shown by significantly enhanced levels of *il1β*, *il6*, *tnfα*, and *ifng1–2* mRNA encoding inflammatory cytokines in R848-treated fish compared with untreated controls (Fig. 5B). However, no significant difference was observed between mutants and wildtype. Next, we investigated the later inflammatory response (8 h posttreatment). We observed a comparable downregulation of mRNA for the inflammatory cytokines *il1β*, *il6*, and *tnfα* in both wildtype and mutant fish compared with the 1-h timepoint. *ifng1–2* displayed a different kinetics, being significantly upregulated in both wildtype and mutant fish still at 8 h (Fig. 5B). Again, this was comparable for wildtype and mutant fish. All together, these data suggest that neither loss of IL-4/13a nor loss of IL-4/13b impairs the response to R848-induced inflammation in the gills.

Next, we evaluated the response to R848 in the absence of both IL-4/13a and IL-4/13b. R848 challenge was performed on the gills of *il4/13a;b^−/−^* double mutants for 1 and 8 h. Significantly enhanced levels of *il1β*, *il6*, *tnfα*, and *ifng1–2* mRNA were observed in the gills of wildtype and *il4/13a;b^−/−^* animals after 1 h. Moreover, levels of *il6* were significantly higher in *il4/13a;b^−/−^* compared with wildtype (Fig. 5C). At 8 h posttreatment, the levels of *il1β*, *il6*, and *tnfα* were reduced almost to baseline in both wildtype and double mutants. However, *ifng1–2* levels were still elevated and again significantly higher in the gills of *il4/13a;b^−/−^* double mutants compared with wildtype (Fig. 5C), suggesting an enhanced type 1 response in agreement with the phenotype observed in the steady state.

### Loss of IL-10 leads to an enhanced inflammatory response to R848 in the gills of il10^e46/e46^ adult zebrafish

**FIGURE 6.**
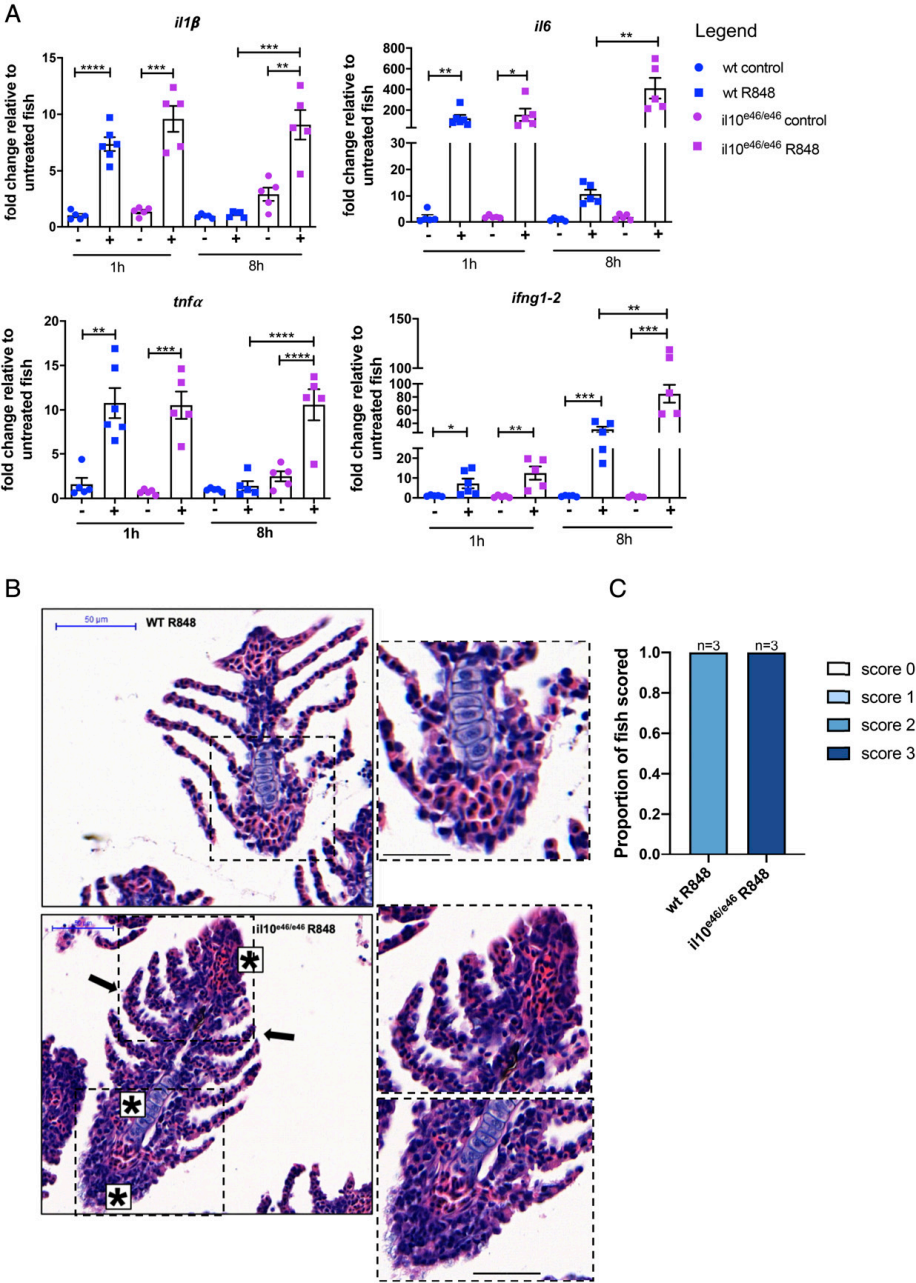
Prolonged inflammatory response in *il10^e46/e46^* gills following R848 stimulation. (**A**) qPCR analysis showing transcript levels of *il1β*, *il6*, *tnfα*, and *ifng1–2* in gills from wildtype and *il10^e46/e46^* animals following R848 challenge for 1 and 8 h. Gene expression was normalized to the expression of β−actin. Fold change is shown. Error bars represent SEM; *n* > 3, *p < 0.05, **p < 0.01, ***p < 0.001, ****p < 0.0001. (**B**) Representative images of sections (*n* = 3) of gills from wildtype and *il10^e46/e46^* animals (*n* = 3) stained with H&E following 8-h R848 challenge. Epithelial cell alteration in the lamellae (black arrows) and gill filament (stars) are indicated in gills from *il10^e46/e46^* animals. Scale bar, 50 μm. (**C**) Quantification of gill tissue damage in wildtype and *il10^e46/e46^* animals following R848 challenge.

Loss of IL-10 resulted in marked alteration of gill morphology in the steady state as shown by the thickening of the gill arch and filaments as well as lamellar epithelial changes (Fig. 4A, 4B) and changes in gene expression indicative of smoldering inflammation (Fig. 4C–E, Table IV). We predicted that inflammatory responses to exogenous irritants would be exaggerated in the absence of IL-10. Therefore, the gills of *il10^e46/e46^* fish were challenged with R848 for 1 and 8 h, as described before. Significant upregulation of *il1β*, *il6*, *tnfα*, and *ifng1–2* mRNA were observed in the gills of stimulated wildtype and *il10^e46/e46^* fish compared with untreated controls after 1 h. However, no difference was observed in the response between wildtype and mutant fish (Fig. 6A), suggesting that loss of IL-10 does not affect the early phase of an inflammatory response. Next, we evaluated the later response (8 h posttreatment), which revealed that compared with wildtype, *il1β*, *il6*, and *tnfα* transcripts were still significantly upregulated in gills from *il10^e46/e46^* animals, and *ifng1–2* transcript levels were even more pronounced (Fig. 6A), indicating a prolonged inflammatory response in the absence of *il10*. In keeping with the exaggerated inflammatory response at 8 h posttreatment, thickening of gill filaments and lamellae were observed in gills from R848-treated *il10^e46/e46^* animals but not wildtype animals (Fig. 6B, 6C). All together, these data suggest that loss of the *il10* gene induced an exaggerated inflammatory response in the gills following R848 stimulation.

## Discussion

In recent years, the field of fish immunology has progressed considerably with many molecules and cell components of both innate and adaptive immune systems having been identified (20, 22, 58). However, our understanding of immune responses, especially in the gills that constitute important mucosal surfaces in fish, is still patchy. In particular, the identity and characteristics of the cells and signals that maintain homeostasis at this mucosal surface have not been fully defined.

In this study, we have used the zebrafish as a model organism to shed light on fish *il4/13a* and *il4/13b* and characterized the consequences of loss of function of these genes under both steady state and inflammatory conditions. Under controlled laboratory conditions, zebrafish lacking IL-4/13A and IL-4/13B were viable, fertile, and did not show any overt phenotype, indicating that such cytokines are not essential for survival and development in zebrafish. Similar findings are observed in IL-4^−/−^ and IL-13^−/−^ mice that are healthy and display no phenotypic abnormalities (59).

We found that disruption of both *il4/13a* and *il4/13b* induced a proinflammatory state in zebrafish larvae, indicating the role for IL-4/13A and IL-4/13B in suppressing inflammation. An anti-inflammatory role for fish type 2 cytokines has been previously demonstrated in trout in which the treatment of head kidney cells with rIL-4/13A and rIL-4/13B2 lead to the downregulation of proinflammatory cytokines (31), resembling the situation in mammals (11). Neutrophils are one of the main components of the zebrafish innate immune system and the first cells to respond to inflammation (60). We found significantly elevated levels of the neutrophil marker *mpx* in *il4/13a;b^−/−^* double mutants, supporting the inflammatory phenotype. The increase in *mpx* transcripts in the *il4/13a^−/−^* single mutants indicates that *il4/13a* may have a potential neutrophil-specific effect albeit one that does not drive a corresponding change in neutrophil number. Effects on *mpx* transcript were not observed in the *il4/13b^−/−^* mutant, suggesting a divergent function for these paralogs in neutrophils in zebrafish. In mammals, neutrophils express IL-4 type I receptor (61, 62), and recent evidence suggests that IL-4– and IL-13–mediated signaling in both mouse and human neutrophils inhibits their migration and effector functions (63, 64) to reduce inflammation and tissue damage during type 2 responses (65). To date, there is no definitive evidence for a role for fish IL-4/13 paralogs in controlling neutrophils. The generation of an *il4/13a;b^−/−^;mpx:EGFP* zebrafish line would be valuable to address neutrophil behavior in the absence of functional IL-4/13A and IL-4/13B.

Previously, we demonstrated that CD4-1^+^ Th2-like lymphocytes resident in the zebrafish gill mucosa were a source of *il4/13b* expression but not *il4/13a* that was detected in gill tissue (37). Recent work has described the presence of ILC-like cells in zebrafish gut, which express high levels of *il4/13a* following helminth Ag exposure, resembling mammalian ILC2 cells (66). In mammals, ILCs contribute to lung homeostasis as well as to lung diseases (67). ILCs might also be present in zebrafish gills, therefore, and be responsible for the expression of *il4/13a*. To shed light on the function of IL-4/13A and IL-4/13B in zebrafish gills, transcriptome analysis was performed in mutants for these cytokines. We found an enrichment of genes implicated in Th1 differentiation and IFN-γ signaling in the gills of *il4/13a;b*^−/−^ double mutants, further supported by qPCR analysis. These data suggest that IL-4/13A and IL-4/13B are jointly required for the maintenance of a Th2-like phenotype in zebrafish gills, and their loss leads to an imbalance toward type 1 immunity. Evidence for IL-4/13 cytokines regulating type 2 immunity was previously reported in fish. In zebrafish, the interaction of IL-4/13A with IL-4Rα on B cell surfaces promoted B cell proliferation and Ab production (68); in trout, treatment of head kidney cells with rIL-4/13 proteins downregulated IFN-γ (31), and upregulation of *il4/13* paralogs was observed in the gills of Atlantic salmon following parasite infection (69).

The transcriptome data predicted a reduction in basophils but an increase in eosinophils in the gills of *il4/13a;b^−/−^* double mutants. In mammals, basophils and eosinophils represent innate sources of IL-4 and IL-13, and their infiltration into tissues is one of the hallmarks of type 2 immune responses (70). Basophils have been described in teleosts (71), and a conserved role for eosinophils was found in response to helminth Ags and infection (72). It might be speculated that decreased basophils and increased eosinophils in the gills of *il4/13a;b^−/−^* double mutants is somehow connected, perhaps reflecting a divergent effect of IL-4/13 on the recruitment of these cell populations to gills. This is unanticipated from mammals in which basophils and eosinophils infiltrate tissues coordinately (73).

The increase in the fraction of M1 macrophages as well as enhanced levels of *tnfα* and *il6* transcripts in the gills of *il4/13a;b^−/−^* double mutants indicate an inflammatory phenotype. Recent studies have identified subsets of polarized macrophages in zebrafish, describing a proinflammatory M1-like phenotype and anti-inflammatory M2-like phenotype, resembling the situation found in mammals (74). Our data suggest that IL-4/13A and IL-4/13B have a conserved function with mammalian IL-4 and IL-13 in driving macrophage polarization as found in other teleost fish (75–78). The increase of IFN-γ in the gills of *il4/13a;b^−/−^* double mutants might also contribute to the induction of M1 macrophages, as previously demonstrated (79), and to the inflammatory phenotype. All together, these findings indicate that loss of IL-4/13A and IL-4/13B leads to an inflammatory phenotype in the gills. This agrees with the phenotype observed in larvae, further supporting an anti-inflammatory function for zebrafish IL-4/13 paralogs.

Despite the induction of proinflammatory signals, loss of IL-4/13A and IL-4/13B did not affect tissue morphology, suggesting that there might be further regulatory mechanisms in place to avoid the disruption of homeostasis in the gills. IL-10 is known to control mammalian immune responses to avoid inflammation and maintain tissue homeostasis. We examined the gills of *il10^e46/e46^* fish and found that IL-10 might be fundamental in maintaining homeostasis in the gills. Transcriptome analysis revealed evidence of smoldering inflammation in the gills of *il10^e46/e46^* fish. In mammals, a lack of IL-10 leads to the development of spontaneous inflammation in the skin, intestine, and lungs (19). In this context, zebrafish IL-10 might have a similar function to its mammalian counterpart.

Recent work has described the use of R848 in inducing inflammation and cytokine response in zebrafish gills (51), highlighting similarities with mouse and human nasal mucosa. In mammals, R848 targets the TLR7 and TLR8 receptors (57), which in fish are mainly expressed in primary lymphoid organs and the gills (80, 81). R848 injection in Atlantic salmon induced IFN expression, whereas stimulation of salmon cell lines failed to induce IFN expression, consistent with the low expression of TLR8 and the lack of expression of TLR7 in these cells (81). These findings suggest that the R848 response in fish might also be mediated by the TLR7/TLR8 pathway, although definitive evidence is lacking. R848 induces a strong inflammatory and IFN response (51, 80). An IFN response is also observed following viral infections in several fish species (82–84), and upregulation of both TLR7 and TLR8 transcripts was detected in infected Atlantic salmon (85). In addition to the IFN response, R848 also induced changes in neutrophil and lymphocyte distribution in zebrafish gills (51). No changes in the distribution but a depletion of large neutrophil-like cells was observed following viral infection in Atlantic salmon (86). Similar to R848 stimulation, an upregulation of proinflammatory cytokines is also observed in response to parasitic infection in salmon and trout (87, 88). In contrast to R848 stimulation, parasitic and bacterial gill infections often lead to major histopathological changes in the tissue (89, 90). This difference might be explained by the acute nature of R848-induced inflammation, which causes only transient effects.

We used R848 to induce inflammation in the gills and found that loss of both IL-4/13A and IL-4/13B led to an enhanced response in the gills of double mutants, which appeared to be type 1 driven, in agreement with transcriptome data in the steady state. In mammals, IL-4 and IL-13 act together to ensure a successful Th2 inflammatory response, and a Th1 phenotype with increased expression of IFN-γ is observed in IL-4/IL-13–deficient mice (59). We infer from our data that in the absence of functional IL-4/13A and IL-4/13B, a Th1-like response dominates in zebrafish, indicating a similar function of these cytokines to their mammalian counterparts.

IL-10 appeared to be fundamental in the maintenance of gill homeostasis, and its loss enhanced the inflammatory response to R848 in the gills, supporting a potent anti-inflammatory function for this cytokine. An anti-inflammatory role for IL-10 has been previously shown in carp (91) and in zebrafish gut (92). Moreover, IL-10–mediated suppression of Th1 cell response and cytokine production was reported in a zebrafish *Mycobacterium marinum* model (56). For the first time, to our knowledge, we addressed the function of zebrafish IL-10 in the gills and demonstrated its importance in preventing inflammation in this mucosal tissue.

Previous studies indicate that fish IL-4/13 paralogs might have divergent functions, with IL-4/13A providing a basal level of type 2 immunity and IL-4/13B required for specific T cell–mediated immunity (31). Our findings indicate that zebrafish IL-4/13 paralogs have a redundant function in suppressing inflammation and maintaining a Th2 phenotype in the gills. Further investigation would be needed to discover unique functions for IL-4/13 cytokines in zebrafish immunity, and our RNA-seq data will be a useful resource in this respect. We propose that the *il4/13a^−/−^*, *il4/13b^−/−^*, and *il4/13a;b^−/−^* zebrafish mutants would be valuable models to investigate the function of *il4/13* paralogs in the context of infection and disease. This would further our knowledge of fish immunology and provide comparative models to answer outstanding questions in mammals.

## Supplementary Material

Data Supplement
