# Supplementary material for: Zebrafish IL-4–like Cytokines and IL-10 Suppress Inflammation but Only IL-10 Is Essential for Gill Homeostasis
Source: J Immunol. 2020 Aug 7;205(4):994–1008. doi: 10.4049/jimmunol.2000372 (PMC7416321; doi:10.4049/jimmunol.2000372)
Supplement: Data Supplement [file JI_2000372.zip › JI_2000372_Supplemental_Figures_1.pdf]

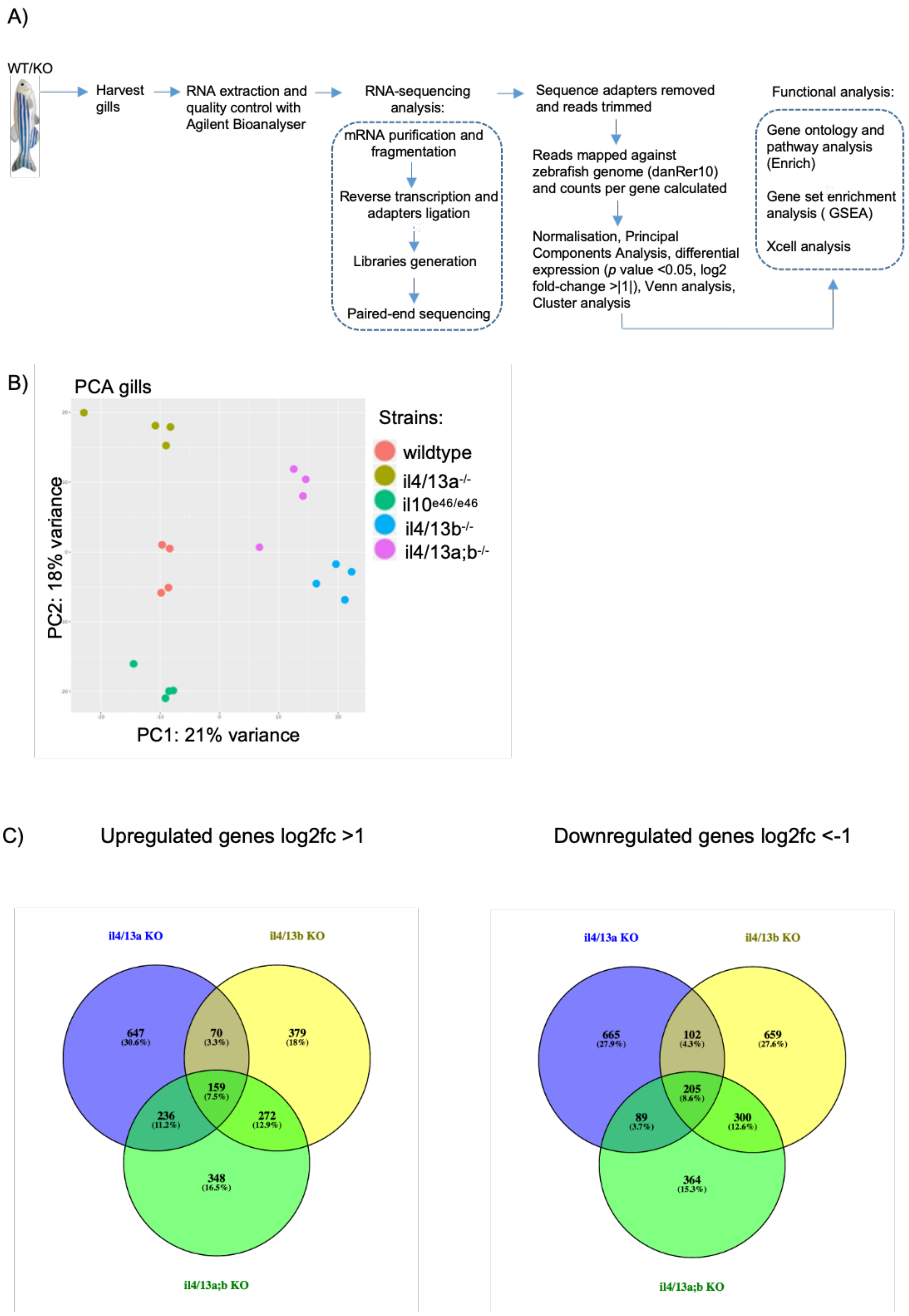

Supplemental Figure 1. (A) RNA-sequencing analysis pipeline. (B) Principal components analysis (PCA) plots representing the distribution of gill samples. (C) Venn diagrams showing the overlap in differentially expressed genes from the gills of *il4/13a*$^{-/-}$, *il4/13b*$^{-/-}$ and *il4/13a;b*$^{-/-}$ animals.

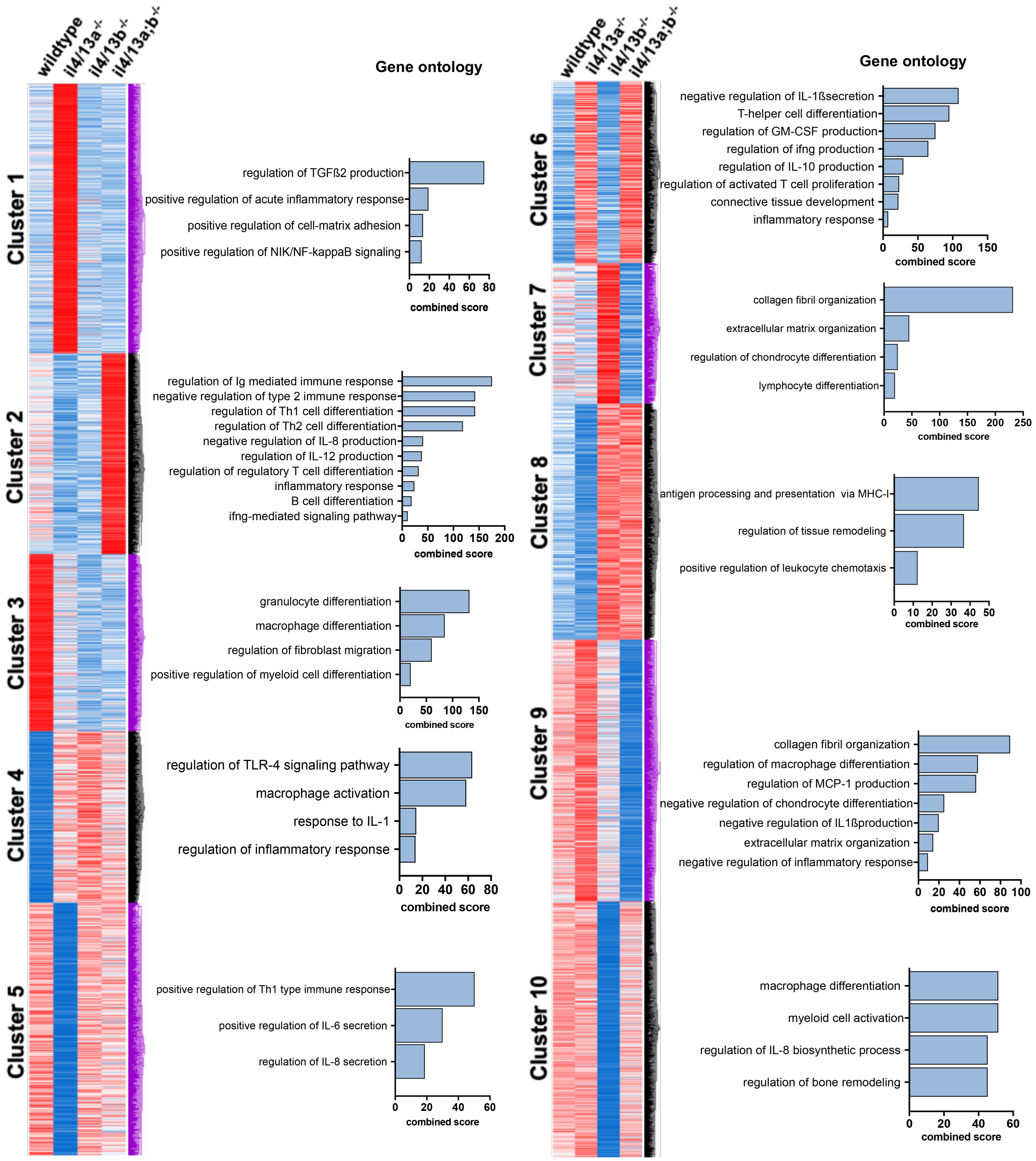

Supplemental Figure 2. Clustering of differentially expressed genes identified in gills harvested from 6-month-old fish. Blue indicates low expression, red indicates high expression and white indicates unchanged expression. Bar graphs show significantly enriched ontologies for each cluster of genes.
